# Ultrasound-Guided Needling Without Lavage Followed by Platelet-Rich Plasma or Corticosteroid in Rotator Cuff Calcific Tendinitis: A Comparative Cohort Study

**DOI:** 10.3390/jcm15155848

**Published:** 2026-07-27

**Authors:** Idris Demirtas, Öner Kilinc, Ali Nadir Tanrikulu, Serhat Elci, Yavuzhan Altin, Fatih Gunaydin

**Affiliations:** 1 Department of Orthopaedics and Traumatology, Mersin City Training and Research Hospital, 33240 Mersin, Turkey; dronerkilinc@hotmail.com (Ö.K.); alimrsntp01@gmail.com (A.N.T.); drfatihgunaydin@gmail.com (F.G.); 2 Department of Orthopaedics and Traumatology, Sultan Private Hospital, 21080 Diyarbakır, Turkey; serhatelci@gmail.com; 3 Department of Orthopaedics and Traumatology, Dr. Ersin Arslan Training and Research Hospital, 27090 Gaziantep, Turkey; dryavuzhanaltin@gmail.com

**Keywords:** shoulder pain, tendinopathy, ultrasonography, treatment outcome, interventional, calcinosis, percutaneous needling

## Abstract

**Background/Objectives**: This study aimed to compare the effects of platelet-rich plasma (PRP) versus corticosteroid injection administered after ultrasound-guided needling without lavage on pain, shoulder function, and radiographic resorption in rotator cuff calcific tendinitis. **Methods**: This retrospective single-centre cohort included 93 patients. All underwent ultrasound-guided needling of the calcific deposit without lavage. Treatment allocation reflected routine clinical practice and was non-randomised. Patients with Gartner type 1–2 calcifications received either PRP or corticosteroid injections, whereas type 3 patients were treated with needling + PRP or conservative management. Outcomes were VAS pain, Constant–Murley Score (CMS), QuickDASH, and radiographic resorption, assessed at baseline, 2 weeks, 6 months, and 12 months. **Results**: In Gartner type 1–2 disease, needling followed by PRP resulted in greater 12-month pain reduction than needling followed by corticosteroid (both *p* < 0.05), while improvements in CMS, QuickDASH, and 1-year complete resorption rates were similar between groups. Gartner type 3 calcifications showed substantial clinical improvement irrespective of treatment modality, but the combined needling + PRP intervention was associated with a higher rate of complete radiographic resorption at 2 weeks than conservative care (72.2% vs. 13.3%; *p* = 0.001). Four patients (4/93, 4.3%) required arthroscopic surgery (three corticosteroid, one PRP). **Conclusions**: In Gartner type 1–2 calcific tendinitis, ultrasound-guided needling followed by PRP was associated with greater 12-month pain reduction than needling followed by corticosteroid, whereas functional and 1-year radiographic outcomes were comparable. In symptomatic Gartner type 3 disease, the combined needling + PRP intervention was associated with faster early radiographic resorption but did not demonstrate superior long-term clinical outcomes; conservative treatment remains appropriate for patients with tolerable symptoms.

## 1. Introduction

Rotator cuff calcific tendinitis (RCCT) is a common cause of shoulder pain and functional limitation, and most patients respond to conservative measures such as rest, NSAIDs, and physiotherapy [1,2]. In cases with persistent symptoms, more-invasive interventions including subacromial injections, ultrasound-guided needle aspiration and lavage (NACD), radiofrequency-based procedures, and extracorporeal shock wave therapy (ESWT) may be considered [3,4,5]. In this context, percutaneous needling without lavage should be distinguished from barbotage/lavage and NACD. In the present study, needling consisted of repeated ultrasound-guided puncture and fragmentation of the calcific deposit without aspiration or saline irrigation, whereas barbotage/lavage and NACD involve fluid-based irrigation and/or aspiration intended to mechanically remove calcific material. Arthroscopic removal of the deposit is reserved for refractory disease [4].

Although NACD has been reported to provide superior outcomes compared with ESWT or isolated corticosteroid injections [6,7], a substantial proportion of patients continue to experience symptoms, and several prognostic factors including female sex, smoking, Gartner type 1 morphology, smaller deposits, and prolonged symptom duration have been associated with poorer outcomes [8,9]. Gartner type 3 calcifications, which generally represent the resorptive phase, are often excluded from NACD studies [9], potentially leading to an underestimation of treatment success despite these lesions being clinically painful and frequently difficult to manage nonoperatively [4,6,10].

Subacromial corticosteroids are commonly administered after NACD to reduce post-procedural inflammation; however, corticosteroids may adversely affect tendon biology and suppress the inflammatory response believed to be essential for subsequent calcific resorption [11,12,13]. Platelet-rich plasma (PRP), in contrast, has emerged as a biological treatment option, with experimental data suggesting enhanced tenocyte proliferation, extracellular matrix synthesis, and angiogenesis [14,15]. Nevertheless, clinical studies investigating PRP in rotator cuff disorders have produced inconsistent results [16,17,18].

Importantly, in most PRP protocols used for calcific tendinopathy, PRP is administered after ultrasound-guided needling and lavage, with the aim of augmenting a biologically mediated healing response in addition to the mechanical clearance of the deposit. A needling-only approach may reduce the confounding influence of mechanical deposit clearance by lavage and thereby allow a more focused comparison between PRP- and corticosteroid-based strategies. Theoretically, avoiding lavage may also preserve local inflammatory mediators and the biologically active microenvironment generated by needle-induced disruption of the deposit, although this hypothesis has not been established clinically. Moreover, recent evidence has questioned the necessity of routine corticosteroid injection after NACD, reporting no clear additional clinical benefit of corticosteroids beyond the effect of the needling–lavage procedure itself [19]. Whether ultrasound-guided needling followed by PRP injection represents a meaningful biological alternative to the more conventional strategy of needling followed by subacromial corticosteroid injection, without formal lavage of the calcific deposit, remains unclear. Because type 3 calcifications represent a distinct resorptive phase with a high spontaneous resolution potential but often substantial symptom burden, their response to intervention may differ from that of denser type 1–2 deposits and therefore warrants separate evaluation. In this phase, local inflammatory activity is thought to contribute to deposit resorption, and corticosteroid-mediated suppression of this response could theoretically interfere with the biological process of calcific resolution [19,20]. Likewise, because Gartner type 3 calcifications are typically excluded from interventional studies, the potential for PRP to accelerate early resorption in this subgroup has not been adequately investigated [20].

Therefore, the aims of this study were

(1) To compare clinical and radiographic outcomes of needling + PRP versus needling + corticosteroid in patients with Gartner type 1–2 RCCT;
(2) To evaluate whether PRP-augmented needling accelerates early radiographic resorption in patients with Gartner type 3 calcifications.

We hypothesised that needling followed by PRP would yield greater long-term clinical improvement than needling followed by corticosteroid injection in Gartner type 1–2 disease and would be associated with faster early radiographic resorption in Gartner type 3 calcifications.

## 2. Materials and Methods

### 2.1. Study Design and Patient Selection

This retrospective cohort study included consecutive patients treated at our institution between June 2018 and January 2024 for symptomatic calcific tendinopathy that had not responded to at least 6 months of conservative management. None of the included patients had undergone previous invasive shoulder intervention or injection for the affected calcific tendinitis before the index treatment. Diagnosis and eligibility were confirmed by clinical examination, standard radiographs, and shoulder magnetic resonance imaging (MRI). All patients underwent MRI before inclusion to assess rotator cuff integrity and to exclude full-thickness rotator cuff tears and other significant structural shoulder pathology.

Inclusion criteria were

- Age ≥ 18 years,
- Symptomatic calcific deposits involving the rotator cuff tendons,
- Radiographic confirmation of calcification,
- Failure of conservative treatment.

Exclusion criteria included

- Full-thickness rotator cuff tears,
- Prior surgery on the affected shoulder,
- Inflammatory arthropathy or coagulopathy,
- Adhesive capsulitis,
- Other significant structural shoulder pathology,
- Patients who were lost to routine outpatient follow-up or had incomplete clinical or radiographic outcome data.

### 2.2. Gartner Classification

Calcific deposits were independently classified as Gartner type 1, 2, or 3 by an experienced radiologist, pain medicine specialist, and orthopaedic surgeon. Disagreements were resolved by consensus. No formal interobserver reliability analysis was performed. Image assessors were blinded to treatment allocation during radiographic evaluation.

### 2.3. Treatment Groups

Treatment selection was based on shared decision-making between the treating physician and the patient. Before treatment, patients were informed about the expected effects, potential benefits, and possible adverse effects of PRP and corticosteroid injection, and the final treatment decision was made jointly. No predefined baseline clinical or radiographic variable was used as an exclusive criterion for assigning patients to PRP or corticosteroid treatment.

Patients were managed using one of three strategies:

1. Needling + PRP injection,
2. Needling + corticosteroid injection,
3. Conservative treatment (Gartner type 3 only).

Gartner type 1–2 patients received either needling + PRP or needling + steroid.

Gartner type 3 patients were managed with needling + PRP or conservative care. Corticosteroid injection was not routinely preferred for Gartner type 3 calcifications in our clinical practice during the study period because of their resorptive-phase biology and high spontaneous resolution potential.

### 2.4. Interventions

#### 2.4.1. Needling + Corticosteroid Protocol

Under ultrasound guidance and local anaesthesia, a 20-gauge needle was advanced into the calcific deposit. Multiple punctures were performed to fragment the calcific material, without saline lavage or aspiration. Thereafter, a subacromial injection of a depot betamethasone preparation (1 mL; betamethasone dipropionate and betamethasone sodium phosphate) mixed with 4 mL of 2% prilocaine was administered under ultrasound guidance around the fragmented deposit and into the subacromial space.

#### 2.4.2. Needling + Platelet-Rich Plasma (PRP) Protocol

PRP was prepared using a commercially available closed preparation kit according to a standardized double-spin centrifugation protocol routinely used at our institution. In brief, 20 mL of autologous venous blood was drawn into tubes containing anticoagulant and processed in two sequential centrifugation steps; after separation of the plasma fraction and a second spin, the platelet-poor supernatant was discarded, and approximately 5 mL of PRP was collected in a sterile syringe. Platelet and leukocyte counts were not routinely measured in the final PRP product. Therefore, the preparation could not be definitively classified as leukocyte-rich or leukocyte-poor, and platelet concentration was not standardized between patients.

Following local anaesthesia, a 20-gauge needle was introduced into the calcific deposit under ultrasound guidance, and multiple punctures were performed to fragment the calcific material without lavage. The freshly prepared PRP was then injected under ultrasound guidance into and around the deposit and into the subacromial space.

All interventional procedures were performed under ultrasound guidance by an experienced pain medicine specialist.

### 2.5. Post-Procedural Care

Patients were discharged after a brief period of observation with standardized post-procedural instructions. Short-term sling immobilization (up to 3 days) was allowed for comfort, followed by gradual progression to active-assisted and active range-of-motion exercises as tolerated. Post-procedural analgesia followed institutional protocols. Patients treated with needling + PRP or needling + corticosteroid were prescribed a fixed-dose tramadol/paracetamol combination (37.5 mg/325 mg) as needed. Patients in the PRP group were specifically instructed to avoid NSAID use during the early post-procedural period. In the conservative group, analgesic treatment consisted of either the same tramadol/paracetamol combination or NSAIDs according to pain severity and clinical need. All patients followed the same standardized post-procedural rehabilitation protocol once pain levels permitted. Rehabilitation was supervised by physiotherapists who were blinded to treatment allocation. Rehabilitation adherence was assessed during routine follow-up visits based on patient-reported compliance and physiotherapy follow-up records.

### 2.6. Outcome Measures

The primary clinical outcome was the change in VAS pain score from baseline to 12 months. Secondary outcomes included CMS, QuickDASH, radiographic resorption, and secondary arthroscopic surgery during follow-up. Clinical outcomes were assessed at baseline and at 2 weeks, 6 months, and 12 months using VAS, CMS, and QuickDASH. Radiographs obtained at baseline, 2 weeks, and 1 year were used to assess changes in calcification size and morphology and complete resorption.

### 2.7. Statistical Analysis

No a priori sample size calculation was performed because of the retrospective design; all eligible consecutive patients meeting the inclusion criteria during the study period were included. No missing outcome data were present in the final analytic cohort; patients with incomplete clinical or radiographic follow-up data were excluded according to the predefined eligibility criteria. Continuous variables were expressed as mean ± standard deviation. Non-normal distributions were analysed using the Kruskal–Wallis test with Holm-adjusted Mann–Whitney U tests for pairwise comparisons. Within-group changes were evaluated using paired Wilcoxon tests. Categorical variables were compared using chi-square or Fisher’s exact tests with Holm correction. Baseline characteristics were compared across the three treatment groups. For clinical outcome changes, between-treatment comparisons were performed separately within each Gartner type: needling + PRP versus needling + corticosteroid for Gartner types 1 and 2, and needling + PRP versus conservative management for Gartner type 3. Effect sizes and 95% confidence intervals were reported when applicable. Significance was set at α = 0.05. Analyses were performed using IBM SPSS Statistics for Windows, version 29.0 (IBM Corp., Armonk, NY, USA).

## 3. Results

**Figure 1:**
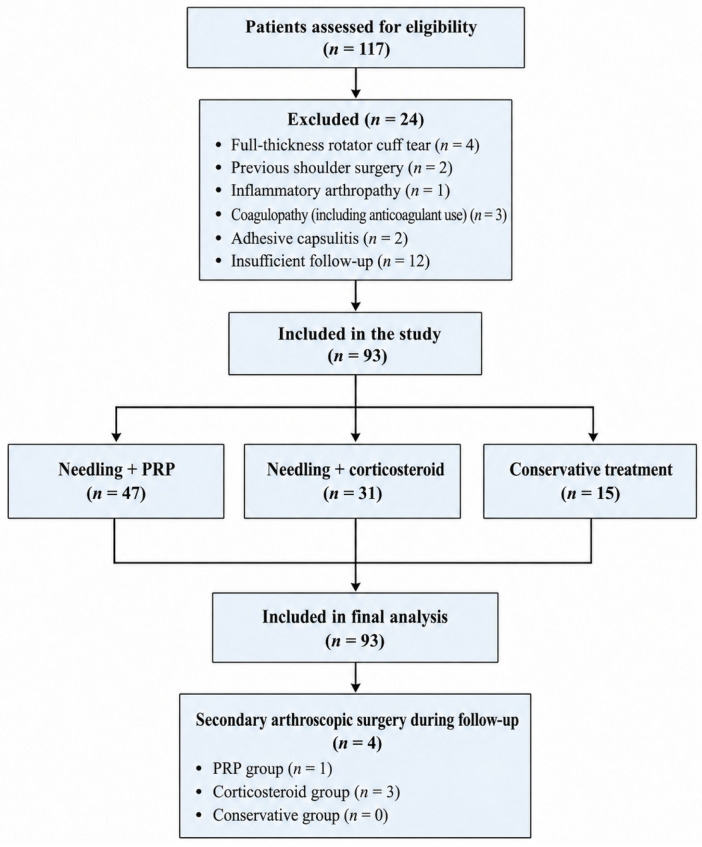
Flow diagram of patient selection, exclusion, treatment allocation, and secondary arthroscopic surgery during follow-up.

A total of 117 patients were assessed for eligibility (Figure 1). Twenty-four patients were excluded because of full-thickness rotator cuff tear (*n* = 4), previous shoulder surgery (*n* = 2), inflammatory arthropathy (*n* = 1), coagulopathy including anticoagulant use (*n* = 3), adhesive capsulitis (*n* = 2), or insufficient follow-up (*n* = 12). The final cohort therefore comprised 93 patients, including 58 women (62.4%) and 35 men (37.6%), with a mean age of 47.9 ± 10.1 years. The dominant shoulder was affected in 65 patients (69.9%). According to the Gartner classification, 31 patients (33.3%) had type 1, 29 (31.2%) had type 2, and 33 (35.5%) had type 3 calcifications. Overall, 47 patients (50.5%) underwent needling + PRP, 31 patients (33.3%) underwent needling + corticosteroid, and 15 patients (16.1%) were managed conservatively. Baseline characteristics according to treatment group are summarized in Table 1.

### 3.1. Baseline Clinical Status

Baseline characteristics according to treatment group are shown in Table 1. Because conservative management was used only in Gartner type 3 disease, baseline VAS and QuickDASH scores differed significantly among treatment groups. Baseline CMS, age, sex distribution, affected side, dominant shoulder involvement, smoking status, type 2 diabetes mellitus, and calcification size were not significantly different among treatment groups. No patient had bilateral involvement. The right side was affected in 55 patients (59.1%) and the left in 38 patients (40.9%). The calcific deposit involved the supraspinatus tendon in 88 patients (94.6%), the infraspinatus tendon in 3 patients (3.2%), and the subscapularis tendon in 2 patients (2.2%). Twenty-seven patients (29.0%) were active smokers, and 7 patients (7.5%) had type 2 diabetes mellitus. Mean calcification size did not differ significantly between treatment groups overall or within each Gartner type. Within Gartner type 1, mean calcification size was 12.6 ± 4.2 mm in the PRP group and 14.1 ± 3.8 mm in the corticosteroid group (*p* = 0.197). Within Gartner type 2, the corresponding values were 13.5 ± 4.1 mm and 14.8 ± 3.9 mm, respectively (*p* = 0.359). In Gartner type 3 disease, mean calcification size was 14.1 ± 4.0 mm in the PRP group and 13.7 ± 3.2 mm in the conservative group (*p* = 0.885).

### 3.2. Radiographic Resorption

At 2 weeks, complete radiographic resorption occurred in

- 0% of type 1,
- 6.9% of type 2,
- 45.5% of type 3 deposits (*p* < 0.001).

By treatment, complete radiographic resorption occurred in

- 27.7% in the PRP group,
- 6.5% in the corticosteroid group,
- 13.3% in the conservative group (*p* = 0.052).
- Among patients with type 3 calcifications, the combined needling + PRP intervention was associated with a significantly higher early radiographic resorption rate than conservative management (72.2% vs. 13.3%; *p* = 0.001).

At 1 year, complete resorption rates remained high and comparable across types (type 1: 77.4%; type 2: 75.9%; type 3: 84.8%; *p* = 0.638) and across treatment modalities (*p* = 0.177).

### 3.3. Pain Outcomes (VAS)

Pain decreased significantly over time in all groups (all *p* < 0.001). Type 3 deposits showed the largest overall reduction (12-month ΔVAS: −5.3 ± 1.4; Table 2).

Within Gartner types 1 and 2, PRP produced significantly greater 12-month pain reduction than corticosteroid:

Type 1: PRP −4.3 ± 1.3 vs. corticosteroid −2.7 ± 1.6; absolute between-group difference in pain reduction, 1.58 points (95% CI, 0.50 to 2.66; *p* = 0.014; Hedges’ g = 1.03).

Type 2: PRP −4.1 ± 1.1 vs. corticosteroid −2.6 ± 1.3; absolute between-group difference in pain reduction, 1.49 points (95% CI, 0.54 to 2.44; *p* = 0.006; Hedges’ g = 1.18).

In type 3, VAS improvement did not differ between PRP and conservative management at any follow-up point.

### 3.4. Functional Outcomes

#### 3.4.1. Constant–Murley Score (CMS)

CMS improved significantly from baseline in all groups (*p* < 0.001). However, no significant between-treatment differences were observed within types 1 or 2 at 6 or 12 months (Table 2). CMS changes in type 3 were also similar between PRP and conservative therapy.

#### 3.4.2. QuickDASH

QuickDASH scores decreased significantly in all types, with the greatest improvements seen in type 3 at both 6 and 12 months (*p* < 0.001). Within each Gartner type, disability reduction did not differ between PRP and corticosteroid treatment (types 1–2) or between PRP and conservative care (type 3) (Table 2).

### 3.5. Secondary Procedures

Four patients (4.3%) underwent arthroscopic surgery during follow-up: three in the corticosteroid group and one in the PRP group. No type 3 patient managed conservatively required surgery (*p* = 0.156). No major procedure-related complications, including deep infection, neurovascular injury, tendon rupture, or clinically significant bleeding, were observed during follow-up.

## 4. Discussion

This study provides two principal findings. First, in patients with Gartner type 1–2 calcifications, ultrasound-guided needling followed by PRP injection resulted in greater long-term pain reduction than needling followed by corticosteroid injection, whereas functional outcomes and 1-year radiographic resorption rates were broadly comparable between the two approaches. Given the inherently subjective nature of pain reporting and the lack of parallel improvements in objective functional scores, the clinical significance of this pain difference should be interpreted with caution. Accordingly, the overall clinical advantage of PRP appears limited primarily to pain reduction rather than a broader improvement in shoulder function. Second, Gartner type 3 calcifications demonstrated substantial clinical improvement irrespective of treatment modality, although the combined needling + PRP intervention was associated with a notably higher rate of early (2-week) complete radiographic resorption than conservative management.

The clinical behaviour of type 3 calcifications observed in this study is consistent with their recognised natural history. These deposits represent the resorptive phase, during which patients frequently experience marked pain but subsequently demonstrate a strong tendency toward spontaneous resolution [7,20,21]. In line with previous prognostic reports, our type 3 cohort exhibited the greatest baseline symptom burden yet showed the most pronounced improvements in pain and shoulder function over time [2,7,20]. The significantly higher early resorption rate in type 3 patients treated with needling + PRP suggests that the combined intervention may accelerate radiographic resolution in selected cases. However, because the interventional group received both needling and PRP whereas the comparator group received conservative management, the present study cannot determine whether the higher early resorption rate was attributable to PRP, the needling procedure, or the combination of both. Moreover, the absence of clinically relevant differences in pain or function at later follow-up indicates that the natural course of the disease remains a major determinant of long-term outcome in this subgroup. The clinical relevance of accelerated radiographic resorption should be interpreted cautiously, as the relationship between structural resolution of calcific deposits and symptomatic or functional improvement has not been consistent across previous studies [6,18,20]. In our type 3 subgroup, **the combined needling + PRP intervention** appeared to accelerate early radiographic clearance of the deposit compared with conservative management, but this advantage did not translate into a meaningful long-term clinical benefit.

Our findings should also be interpreted in the context of previous studies evaluating PRP in calcific tendinopathy. Oudelaar et al. reported no consistent superiority of PRP over corticosteroid when both were administered after NACD [18]. In contrast, in the present cohort both PRP and corticosteroid were administered after ultrasound-guided needling of the calcific deposit without lavage, allowing a direct comparison between a biological injectate and a conventional corticosteroid injection protocol in a purely needling-based setting. Taken together, these observations also suggest that, irrespective of the injectate used, ultrasound-guided needling without lavage may itself contribute meaningfully to promoting calcific resorption and improving shoulder function.

A randomized trial comparing saline and corticosteroid injections after NACD found that non-inferiority of saline to corticosteroid could not be demonstrated. Corticosteroid injection was associated with greater pain relief in the first 6 weeks and improved function at 3 months, while having no meaningful impact on the degree of calcific deposit resorption [19]. Together with concerns regarding the potentially detrimental effects of corticosteroids on tendon integrity, these observations provide a biological rationale for further investigation of PRP as a potential alternative after needling in selected patients with chronic Gartner type 1–2 disease [22].

The broader PRP literature in rotator cuff-related pain has commonly demonstrated a pattern of slower but more-durable symptom relief compared with corticosteroids, whereas differences in functional recovery tend to diminish over time [23,24,25]. This trajectory is consistent with our results: in Gartner type 1–2 disease, needling followed by PRP yielded greater long-term improvement in pain without substantial additional gains in CMS or QuickDASH compared with needling followed by corticosteroid injection. The dissociation between pain and functional outcomes may reflect a ceiling effect of rehabilitation and the primarily biological, rather than mechanical, impact of PRP. A plausible explanation for the greater long-term pain reduction observed after PRP is the activity of platelet-derived growth factors and other bioactive mediators, which may support tendon remodelling and modulate persistent local inflammation. However, this mechanism remains speculative in the present study because PRP composition was not quantified and no biological biomarkers were assessed.

In our clinical setting, the direct treatment cost of PRP was several-fold higher than that of corticosteroid injection because of the additional preparation materials, centrifugation, and consumables required. This greater resource burden should be taken into account when interpreting the overall clinical value of PRP.

Although RCCT is generally considered a self-limiting condition, symptom resolution may be prolonged, and the disease can substantially affect pain, sleep, daily activities, and quality of life. Interventional treatment may therefore be considered in patients with persistent symptoms despite prolonged conservative management or in those seeking more-rapid symptom improvement. These potential benefits should nevertheless be balanced against procedure-related discomfort, small risks of bleeding, infection, and local tissue irritation, as well as the additional burden and cost associated with PRP.

Several limitations warrant consideration. The retrospective, single-centre design and absence of randomisation introduce a risk of selection bias. No formal sensitivity analysis, propensity-score adjustment, or other statistical method specifically designed to address treatment-selection bias was performed. Therefore, residual confounding related to the non-randomised treatment allocation cannot be excluded. Although Holm correction was applied for pairwise comparisons, the analysis included multiple outcomes and subgroup comparisons; therefore, a residual risk of type I error cannot be completely excluded. Sample sizes within the combined Gartner-treatment subgroups were modest, limiting the statistical power to detect smaller between-group differences. Work or sports activity level and detailed information regarding previous physiotherapy exposure were not consistently available in the retrospective records. PRP composition was not quantitatively characterised, and platelet and leukocyte concentrations were not standardized between patients. This limits direct comparison with previous studies using different PRP formulations and reduces the reproducibility of the present protocol across clinical settings. Although the higher direct resource burden of PRP was acknowledged, no formal cost-effectiveness analysis was performed. Radiographic follow-up relied on plain radiographs rather than ultrasound, which may have underestimated partial resorption and subtle changes in deposit morphology. Finally, participants and clinical outcome assessors were not blinded to treatment allocation, although image assessors were blinded during radiographic evaluation.

Despite these limitations, the study has several strengths, including the use of standardised ultrasound-guided techniques, the inclusion and separate analysis of all three Gartner types, and comprehensive longitudinal assessment of pain, function, and radiographic change. Clinically, the results suggest that, in selected patients with Gartner type 1–2 calcific tendinitis, ultrasound-guided needling followed by PRP may provide greater long-term pain reduction than needling followed by corticosteroid injection, although functional and radiographic outcomes appear comparable. In symptomatic Gartner type 3 disease, the combined needling + PRP intervention may be considered for patients with substantial discomfort who desire more-rapid radiographic resolution, whereas conservative management remains appropriate for those with tolerable symptoms, given the generally favourable natural history.

## 5. Conclusions

In patients with Gartner type 1–2 calcific tendinitis, ultrasound-guided needling followed by PRP was associated with greater 12-month pain reduction than needling followed by corticosteroid injection. However, functional outcomes and 1-year radiographic resorption were comparable between groups, and the present study was not designed to determine whether the observed between-group differences in VAS exceeded a prespecified threshold for clinical importance. Therefore, these findings should not be interpreted as demonstrating overall clinical superiority of PRP. In Gartner type 3 disease, the combined needling + PRP intervention was associated with faster early radiographic resorption than conservative management, but this advantage did not translate into superior long-term pain or functional outcomes. Treatment decisions should therefore consider symptom severity, patient preferences, procedural burden, cost, and the natural history of the disease. Randomized controlled trials using standardized and quantitatively characterized PRP preparations are required to confirm these findings.

## Figures and Tables

**Table 1:** Baseline characteristics according to treatment group.

Variable	N + PRP (*n* = 47)	N + CS (*n* = 31)	Conservative (*n* = 15)	*p* Value
Age, years	48.5 ± 10.3	47.8 ± 9.3	46.1 ± 11.1	0.754
Female sex	29 (61.7%)	19 (61.3%)	10 (66.7%)	0.931
Right side	27 (57.4%)	18 (58.1%)	10 (66.7%)	0.810
Dominant shoulder involvement	33 (70.2%)	21 (67.7%)	11 (73.3%)	0.926
Calcification size, mm	13.4 ± 4.0	14.4 ± 3.8	13.7 ± 3.2	0.459
Baseline VAS	6.3 ± 1.1	5.2 ± 1.2	6.9 ± 1.2	<0.001
Baseline CMS	47.9 ± 10.3	50.6 ± 9.9	44.4 ± 14.0	0.285
Baseline QuickDASH	56.0 ± 14.4	46.0 ± 14.8	65.4 ± 8.6	<0.001
Gartner type				
Type 1	14 (29.8%)	17 (54.8%)	0 (0.0%)	--
Type 2	15 (31.9%)	14 (45.2%)	0 (0.0%)	--
Type 3	18 (38.3%)	0 (0.0%)	15 (100.0%)	--

Values are presented as mean ± SD or number (%), as appropriate. N + PRP: needling + platelet-rich plasma; N + CS: needling + corticosteroid; VAS: visual analogue scale; CMS: Constant–Murley Score; QuickDASH: shortened Disabilities of the Arm, Shoulder and Hand questionnaire; PRP: platelet-rich plasma; SD: standard deviation. Gartner type distribution differed by study design because conservative management was used only in patients with Gartner type 3 calcifications. “--” indicates that no statistical comparison was performed because Gartner type distribution was determined by the study design.

**Table 2:** Changes in clinical outcome scores according to Gartner type and treatment group.

Parameters	Gartner Type	N + PRP Mean ± SD	N + CS Mean ± SD	Conservative Mean ± SD	*p* Value
ΔVAS at 2 weeks	Type 1 Type 2 Type 3	−0.9 ± 1.4 −2.1 ± 1.6 −3.2 ± 2.0	−0.8 ± 1.7 −1.1 ± 1.9 --	-- -- −3.1 ± 1.7	0.871 0.195 0.985
ΔVAS at 6 months	Type 1 Type 2 Type 3	**−3.9 ± 1.5** −3.5 ± 1.5 −4.6 ± 1.8	**−2.2 ± 1.6** −2.3 ± 1.6 --	-- -- −5.0 ± 1.4	**0.025** 0.062 0.530
ΔVAS at 12 months	Type 1 Type 2 Type 3	**−4.3 ± 1.3** **−4.1 ± 1.1** −4.8 ± 1.4	**−2.7 ± 1.6** **−2.6 ± 1.3** --	-- -- −5.3 ± 1.4	**0.014** **0.006** 0.346
ΔCMS 6 months	Type 1 Type 2 Type 3	+7.5 ± 11.7 +11.3 ± 15.0 +29.9 ± 16.7	+6.1 ± 13.6 +14.4 ± 14.8 --	-- -- +22.4 ± 16.5	0.937 0.677 0.175
ΔCMS 12 months	Type 1 Type 2 Type 3	+34.1 ± 7.9 +37.1 ± 8.4 +43.6 ± 11.7	+37.3 ± 10.5 +30.9 ± 12.8 --	-- -- +41.2 ± 16.0	0.381 0.182 0.842
ΔQuickDASH 6 months	Type 1 Type 2 Type 3	−9.0 ± 16.1 −21.3 ± 18.3 −31.9 ± 12.5	−16.2 ± 22.7 −17.7 ± 17.4 --	-- -- −33.8 ± 15.2	0.258 0.585 0.600
ΔQuickDASH 12 months	Type 1 Type 2 Type 3	−32.2 ± 18.7 −35.3 ± 14.4 −55.1 ± 10.2	−25.3 ± 19.2 −28.4 ± 14.7 --	-- -- −56.3 ± 9.9	0.372 0.169 0.504

Values are presented as mean ± SD. SD: standard deviation; VAS: visual analogue scale; CMS: Constant–Murley Score; QuickDASH: shortened Disabilities of the Arm, Shoulder and Hand questionnaire; PRP: platelet-rich plasma; CS: corticosteroid. N + PRP: needling + platelet-rich plasma; N + CS: needling + corticosteroid. *p* values indicate between-treatment comparisons within each Gartner type. Bold values indicate statistical significance (*p* < 0.05). “--” indicates that the treatment group was not applicable for that Gartner type.

## Data Availability

The data supporting the findings of this study are not publicly available due to privacy and ethical restrictions related to patient confidentiality. De-identified data may be made available from the corresponding author upon reasonable request and with permission of the relevant institutional ethics committee.

## Abbreviations

The following abbreviations are used in this manuscript:

RCCT	Rotator cuff calcific tendinitis
PRP	Platelet-rich plasma
NACD	Needle aspiration of calcific deposits
N + PRP	Ultrasound-guided needling without lavage followed by platelet-rich plasma injection
N + CS	Ultrasound-guided needling without lavage followed by corticosteroid injection
VAS	Visual analogue scale
CMS	Constant–Murley Score
QuickDASH	Quick Disabilities of the Arm, Shoulder and Hand
ESWT	Extracorporeal shock wave therapy
MRI	Magnetic resonance imaging
